# Petroleum Pitch-Derived Porous Carbon Materials as Metal-Free Catalyst for Dry Reforming of Methane

**DOI:** 10.3390/molecules29194642

**Published:** 2024-09-29

**Authors:** Kaixuan Huo, Yu Sun, Hu Jiang, Shiyuan Lin, Haiqiu Fang, Zhinian Cheng, Shaochen Cao, Liangjun Li, Yang Wang, Mingbo Wu

**Affiliations:** College of New Energy, College of Chemistry and Chemical Engineering, State Key Laboratory of Heavy Oil Processing, China University of Petroleum (East China), Qingdao 266580, China; s22030129@s.upc.edu.cn (K.H.); s23150055@s.upc.edu.cn (Y.S.); z21030096@s.upc.edu.cn (H.J.); bz23150003@s.upc.edu.cn (S.L.); haiqiufang@upc.edu.cn (H.F.); chengzhinian02@163.com (Z.C.); curtainsc17@163.com (S.C.); lilj@upc.edu.cn (L.L.)

**Keywords:** porous carbon materials, metal-free catalyst, dry reforming of methane, ketone/quinone carbonyl groups, surface area

## Abstract

Porous carbon materials have gained increasing attention in catalysis applications due to their tailorable surface properties, large specific surface area, excellent thermal stability, and low cost. Even though porous carbon materials have been employed for thermal-catalytic dry reforming of methane (DRM), the structure–function relationship, especially the critical factor affecting catalytic performance, is still under debate. Herein, various porous carbon-based samples with disparate pore structures and surface properties are prepared by alkali (K_2_CO_3_) etching and the following CO_2_ activation of low-cost petroleum pitch. Detailed characterization clarifies that the quinone/ketone carbonyl functional groups on the carbon surface are the key active sites for DRM. Density functional theory (DFT) calculations also show that the C=O group have the lowest transition state energy barrier for CH_4_* cleavage to CH_3_* (2.15 eV). Furthermore, the cooperative interplay between the specific surface area and quinone/ketone carbonyl is essential to boost the cleavage of C-H and C-O bonds, guaranteeing enhanced DRM catalytic performance. The MC-600-800 catalyst exhibited an initial CH_4_ conversion of 51% and a reaction rate of 12.6 mmol_CH4_ g_cat._^−1^ h^−1^ at 800 °C, CH_4_:CO_2_:N_2_= 1:1:8, and GHSV = 6000 mL g_cat._^−1^ h^−1^. Our work could pave the way for the rational design of metal-free carbon-based DRM catalysts and shed new light on the high value-added utilization of heavy oils.

## 1. Introduction

Methane is an ideal resource for hydrogen or syngas (CO + H_2_) production via catalytic technology such as steam reforming of methane (SRM), partial oxidation of methane (POM), dry reforming of methane (DRM), and catalytic methane decomposition (CMD) [1,2,3,4]. Among all the catalytic methane transformation strategies, DRM, utilizing two greenhouse gases (CH_4_ + CO_2_) to produce high-quality syngas (CO/H_2_), possesses the integrated functions of greenhouse gas elimination and valuable chemical supply. The DRM product, namely syngas with a molar ratio of 1, can be employed as an ideal feedstock for liquid fuels or light olefin production via Fischer–Tropsch synthesis technology [5,6]. Even though DRM is gaining more and more attention from industry and academia due to its large-scale application potential and environmental benefits, the absence of stable and effective catalysts leads to an absence of commercially feasible technology [7]. Therefore, the current interest in DRM is mainly focused on the development of suitable catalysts and optimizing their catalytic stability. Various transition metals such as Ni, Co, and Pd have been explored as active catalysts for DRM, but most of them suffer from high cost or rapid deactivation because of sintering at high reaction temperatures as well as carbon deposition. Therefore, the search for DRM catalysts with excellent catalytic performance and appropriate economic cost for large-scale utilization is highly urgent [8].

Metal-free carbon-based catalysts stand out among various candidates regarding high-temperature sintering/carbon deposition-resistance and low cost [9]. Firstly, the pure carbon-based catalysts without doping from metallic active sites could effectively avoid the undesirable active sites sintering, especially under high reaction temperatures. Furthermore, deactivation by carbon deposition is not problematic for DRM, as the deposited carbon can act as a new catalyst for the subsequent DRM reaction, as reported in other literature, thus improving the activity in the later stage [10]. So far, many researchers have evaluated the DRM performance of various carbon materials, such as activated carbon (AC), carbon black (CB), carbon nanotubes (CNT), ordered mesoporous carbon materials, etc. Li et al. [11] found that coal char could exhibit DRM catalytic activity, and its specific surface area and ash content are two key factors affecting the DRM performance. Petroleum asphalt-based carbon materials also showed appealing activity and stability for DRM [12]. It is worth noting that the application of coal char and petroleum asphalt-based carbon materials in DRM are attractive due to the realization of the value-added utilization of low-cost resources. Suelves et al. [13] reported a linear relationship between methane conversion and the number of oxygen-containing functional groups on the carbon surface by measuring the desorption of CO and CO_2_ during thermal treatment. Even though tremendous progress has been made in carbon-based DRM catalyst exploration and active site identification, the structure–function relationship of the carbon-based catalysts has not been well defined, leading to difficulties in understanding the catalytic mechanism and to further enhance DRM catalytic performance.

To tackle the above-mentioned obstacles, this study aims at optimizing the content of the oxygen-containing functional groups and architectural properties of the petroleum pitch-derived porous carbon materials by tuning the K_2_CO_3_ activation temperature and the subsequent CO_2_ activation atmosphere. Additionally, the DRM reaction is used as a model to investigate the influence of oxygen-containing functional groups on the catalyst surface in activating C-H bonds. Three discrete oxygen-containing functional groups can be identified by deconvolution of the O1s peak of X-ray photoelectron spectroscopy (XPS): C=O group, O=C-O group, and C-OH/C-O-C group. Based on experimental and characterization results, the role of the quinone/ketone carbonyl oxygen-containing functional groups (C=O) in DRM is identified. Density functional theory (DFT) calculations also show that C=O groups play a crucial role in promoting the cleavage of CH_4_. Furthermore, CO_2_ activation can increase the specific microporous surface area and the accessibility of reactants to the catalytic interface without losing the number of essential active sites. This would further enhance the DRM performance of the carbon-based catalyst. This study contributes to the in-depth understanding of how specific oxygen-containing functional groups affect the activity of porous carbon materials during catalytic processes, which not only enriches the understanding of the activity nature of carbon-based catalysts in DRM reactions but also provides an essential theoretical basis for the design of novel and efficient carbon-based catalysts.

## 2. Results and Discussion

### 2.1. Preparation and Structural Characterization of K_2_CO_3_-Activated Catalysts

**Figure 1:**
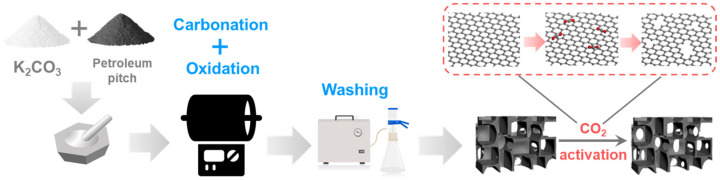
Schematic illustration of the preparation procedure of metal-free catalyst (MC-T, T denotes the CO_2_ activation temperature, °C).

The porous carbon materials catalysts were prepared by the K_2_CO_3_ chemical activation method. The preparation scheme is shown in Figure 1. According to the research literature, the mechanism of preparing porous carbon materials by activation with K_2_CO_3_ at high temperatures includes the following chemical reactions (Equations (1)–(4)) [14]:

(1) $$K_{2}{\mathrm{CO}}_{3}\to K_{2}O+{\mathrm{CO}}_{2}$$

(2) $$K_{2}{\mathrm{CO}}_{3}+2C\;\to \;2K+3\mathrm{CO}$$

(3) $$K_{2}O+C\;\to \;2K+\mathrm{CO}$$

(4) $${\mathrm{CO}}_{2}+C\;\to \;2\mathrm{CO}$$

**Figure 2:**
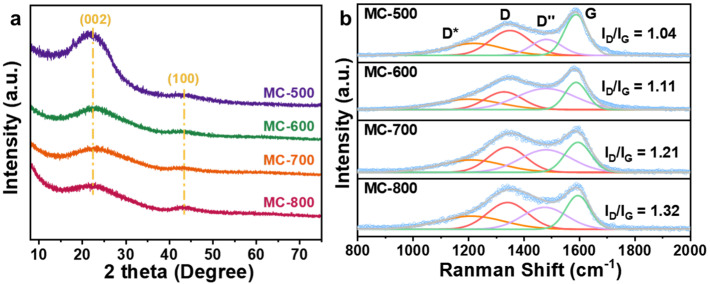
(**a**) XRD patterns and (**b**) Raman spectra of MC-500/600/700/800 catalysts.

The XRD patterns of the MC-T catalysts are displayed in Figure 2a. All samples exhibited two broad peaks at 24° and 43°, which can be attributed to the disorder graphite structure of crystal planes (002) and (100) (0.34 nm and 0.21 nm interplanar distances, respectively) [15,16]. The (002) peak is a standard characteristic of the interlayer stacking structure of the conjugated aromatic system, which indicates the graphite-like structure of the porous carbon materials catalysts. Compared with the standard diffraction peak position (26°), the diffraction peak shifted to a lower angle. According to the Bragg diffraction formula [17] (d = nλ/2sin θ, where d is the inter planar spacing, 2θ is the diffraction peak angle, n = 1, λ = 1.5406 Å), the activation of K_2_CO_3_ increased the carbon layer spacing. With the increase with activation temperature during the preparation process, the diffraction peak widened, and the intensity decreased, indicating an increase in the amorphous degree of the porous carbon materials.

Figure 2b shows the Raman spectra and the fitted compositions of the MC-T catalysts. In addition, the D peak (near 1350 cm^−1^) and G peak (near 1580 cm^−1^) can be attributed to the defective carbon and graphitic carbon, respectively [18]. In addition, there are also D* peaks located near 1200 cm^−1^ and D″ peaks near 1450 cm^−1^, attributed to the vibration of C-C and C=C bonds of polyenic compounds, respectively, which is typical for annealed and activated organic structures [19,20]. Notably, The I_D_/I_G_ values increased as the carbonization temperature increased (1.04, 1.11, 1.21, 1.32 for MC-500/600/700/800/, respectively), indicating that the defect degree of the catalysts increased with the increase in carbonization temperature. The imperfect structures, such as defects, edges, and vacancies, could lead to the localization of π electrons and promote the generation of oxygen-containing groups on the surface, therefore altering the catalytic performance.

**Figure 3:**
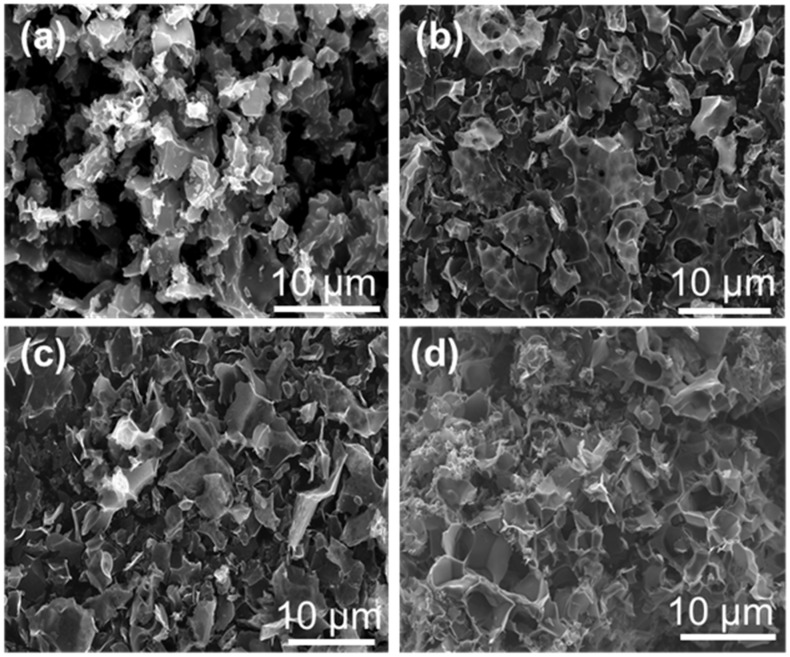
SEM images of (**a**) MC-500, (**b**) MC-600, (**c**) MC-700, and (**d**) MC-800 catalysts.

The effect of the K_2_CO_3_ activation temperature on the catalyst morphology was further monitored by scanning electron microscope (SEM) (Figure 3). The SEM images revealed that all the porous carbon-based catalysts presented a three-dimensional lamellar porous structure. With the increase in activation temperature, the carbon flakes became more dispersed. This structural change was conducive to the exposure of catalytically active sites and mass transfer behavior during the reaction process, thus accelerating the diffusion and adsorption of the reactants and improving their conversion rate.

### 2.2. Structure–Function Relationship of the K_2_CO_3_-Activated Catalysts

**Figure 4:**
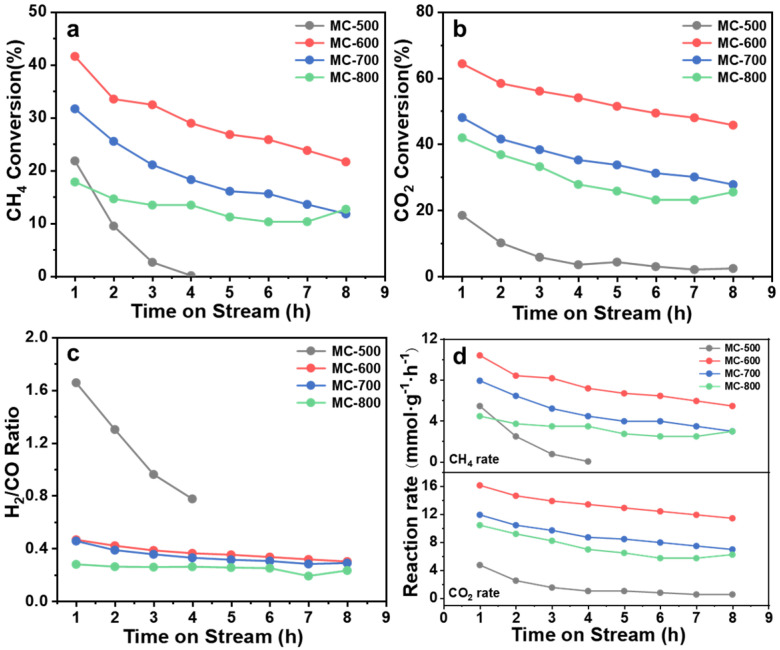
(**a**) CH_4_ conversion, (**b**) CO_2_ conversion, (**c**) H_2_/CO ratio and (**d**) Reaction rate over reaction time of MC-500/600/700/800 catalysts. Reaction conditions: GHSV = 6000 mL g_cat._ ^−1^ h^−1^; P = 1 atm; CH_4_: CO_2_ = 1:1; temperature: 800 °C.

The DRM reaction of K_2_CO_3_-activated catalysts was performed at 800 °C (Figure 4). The conversion of CO_2_ and CH_4_ on MC-600 was much higher than that of MC-500/700/800, reaching a maximum of 64.32% and 50.66%, respectively. The corresponding initial reaction rates were also the highest, reaching 10.39 mmol_CH4_ g_cat._^−1^ h^−1^ and 16.08 mmol_CO2_ g_cat._^−1^ h^−1^, respectively. The highest conversion may result from more oxygen-containing functional groups on MC-600. However, all catalysts had poor stability, with the CO_2_ and CH_4_ conversion decreasing significantly during the 8 h test. This phenomenon may be attributed to carbon deposition during the reaction process, preventing the reactants from reaching the active site [21]. Notably, as illustrated in Figure 4, the CO_2_ conversion consistently surpassed the CH_4_ conversion, indicating that the reverse water-gas shift reaction (RWGS: CO_2_ + H_2_ → CO + H_2_O) occurred to a certain extent. For the MC-600/700/800 catalysts, the molar ratio of H_2_/CO was between 0.23 and 0.46 during the catalytic performance test, below the theoretical value of 1.0. Moreover, the H_2_/CO ratio gradually declined while the reaction time evolved, indicating that the RWGS reaction intensified [22]. Conversely, the H_2_/CO ratio from the MC-500 catalyst exceeded 1.0, potentially due to the thermal decomposition of the catalyst under a high reaction temperature.

**Figure 5:**
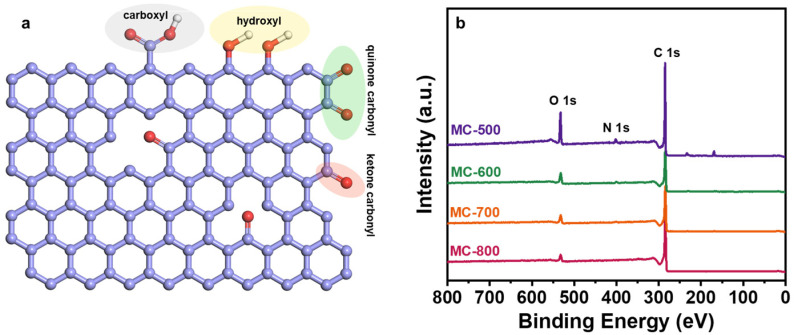
(**a**) Oxygen-containing functional groups on the carbon surface and (**b**) XPS survey spectra of MC-500/600/700/800 catalysts.

**Figure 6:**
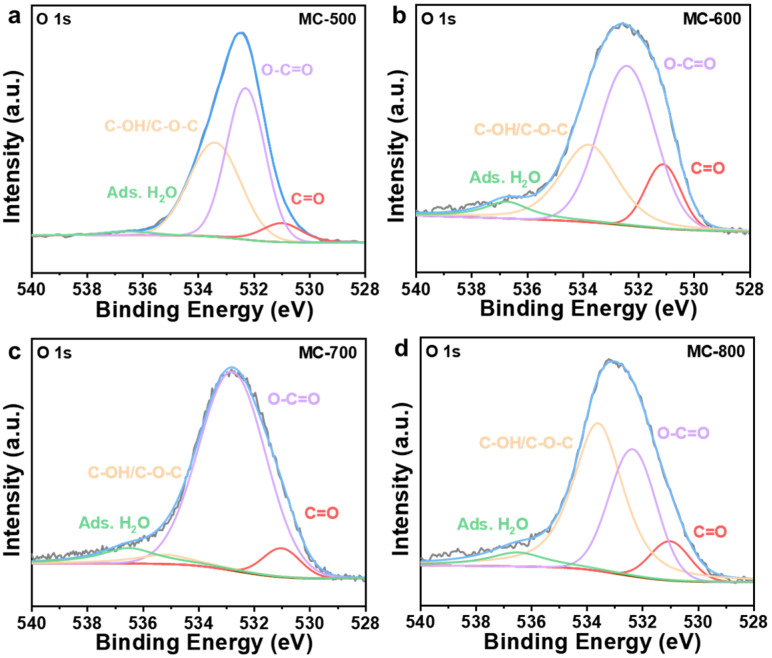
High-resolution O 1s XPS spectra of (**a**) MC-500, (**b**) MC-600, (**c**) MC-700, and (**d**) MC-800 catalysts.

**Figure 7:**
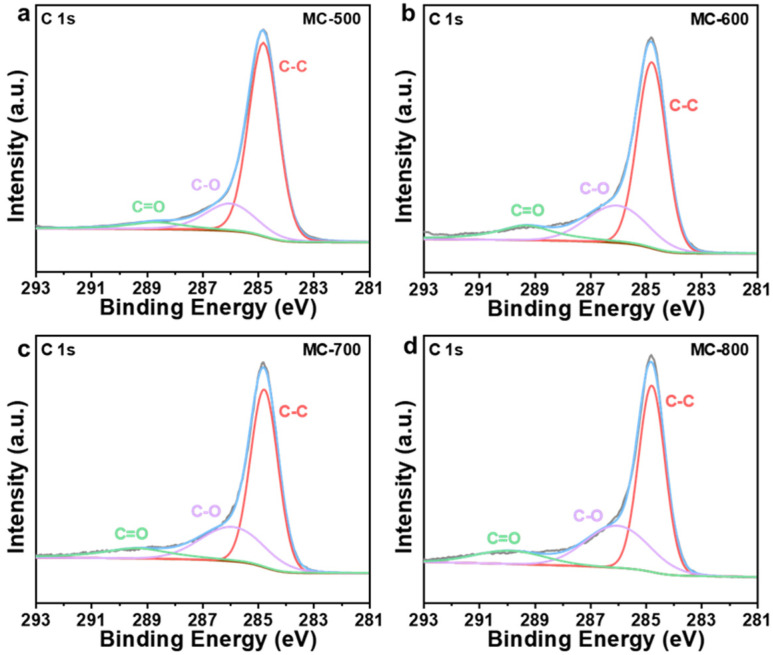
High-resolution C 1s XPS spectra of (**a**) MC-500, (**b**) MC-600, (**c**) MC-700, and (**d**) MC-800 catalysts.

Several researchers have reported that oxygen-containing functional groups can be employed as active sites of metal-free carbon catalysts, especially in dehydrogenation reactions [23,24]. Oxygen-containing functional groups could tailor the electronic properties of the porous carbon materials catalysts, thus optimizing the relevant catalytic reactions. Typical oxygen-containing functional groups, such as carboxyl (O=C-O), quinone/ketone carbonyl (C=O), and hydroxyl (C-OH) on carbon materials are shown in Figure 5a. According to the X-ray photoelectron spectroscopy (XPS) results shown in Figure 5b, the porous carbon materials catalyst was mainly composed of C (284.3 eV) and O (531.9 eV) elements. At the same time, a trace amount of N with XPS binding energy at 399.9 eV was also detected in the porous carbon materials, which can be derived from the petroleum pitch precursor [25]. Notably, the MC-500 samples also contained trace amounts of S (S 2p, 165 eV; S 2s, 225 eV), which may be attributed to the incomplete decomposition of the S-containing groups in the petroleum pitch precursor under lower calcination temperatures [26,27]. Moreover, with the increase in activation temperature, the ratio of N/C content (0.09, 0.05, 0.02, 0.01 for MC-500/600/700/800/, respectively) and the ratio of O/C content (0.48, 0.31, 0.24, 0.17 for MC-500/600/700/800/, respectively) decreased gradually, which indicates that the N- and O-containing groups could be decomposed at high temperatures. The oxygen-containing functional groups were further characterized by deconvolution of the XPS O 1s spectra (Figure 6). The peaks can be attributed to C=O (531.0–531.8 eV), O=C-O (532.2–532.8 eV), C-OH/C-O-C (533.5 eV), and adsorbed water (535.0–536.2 eV), respectively [28]. The relative concentration of oxygen-containing groups was quantified using the peak area fitting method (Table 1). The MC-600 catalyst possessed the highest content of the C=O group, which is consistent with the results of the C 1s XPS analysis (Figure 7 and Table 2) [29]. Considering the superior CH_4_ conversion of the MC-600 catalyst, the C=O group may be the essential active site for C-H bond cleavage during DRM reaction [30]. As reported in other literature, C-H bonding in CH_4_ molecule could activate the C=O group on the porous carbon materials surface [31]. Therefore, it is reasonable to believe that the C=O active sites on porous carbon materials could deliver the C-H bond cleavage function via the C=O → C-OH → C=O pathway. In addition, O=C-O, C-OH, and other acidic oxygen-containing groups have been widely reported as the active sites of cracking side reactions [32]. Typically, a lower activation temperature is favorable for the formation of oxygen-containing functional groups. However, as the activation temperature increases, oxygen atoms are inclined to be removed and converted to gaseous oxides [33]. Therefore, 600 °C is the optimum activation temperature with the most active sites for C-H bond cleavage and the least active sites for cracking side reactions.

**Figure 8:**
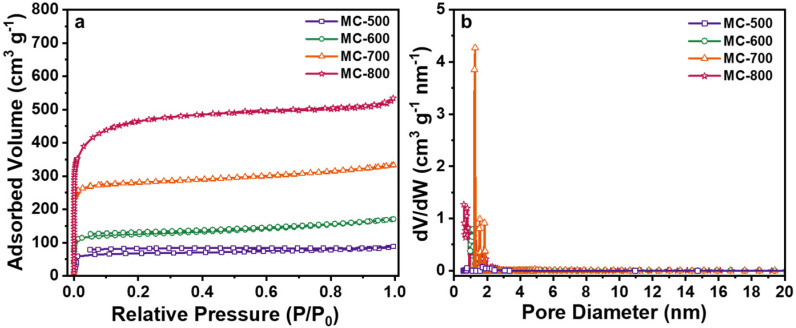
(**a**) N_2_ adsorption/desorption isotherms and (**b**) Pore size distribution curves of MC-500/600/700/800 catalysts.

N_2_ adsorption/desorption isotherms were performed to analyze the specific surface area and pore size of the porous carbon materials (Figure 8). All of the catalysts displayed typical I isotherms, indicating that the porous carbon materials catalysts prepared by chemical K_2_CO_3_ activation were mainly microporous structures and had a narrow pore size distribution centered at 1.2 nm [34]. As expected, with the increase in activation temperature, the N_2_ adsorption capacity of the catalyst increased obviously, indicating that the increase in activation temperature could significantly enhance the pore structure of the catalyst, which can be attributed to the stronger etching capability of K_2_CO_3_ under high temperatures. As listed in Table 3, the porous carbon materials catalysts exhibited markedly different total specific surface areas (S_BET_), ranging from 214 m^2^ g^−1^ to 1748 m^2^ g^−1^. With the increase in activation temperature, the S_BET_ and the total pore volume (V_t_) increased mainly in the microporous specific surface area (S_mir_) and microporous volume (V_mir_). In contrast, the pore size (D_AVE_) first increased and then decreased. This could be attributed to the increased microporous collapse and reduced pore size when the calcination temperature exceeded 700 °C [35]. Notably, no positive correlation between the architectural properties and the DRM catalytic performance was detected from the K_2_CO_3_-activated catalysts. However, theoretically, advanced structure properties, such as high specific surface area and large pore size, are favorable to speed up the transportation of reactant molecules and increase the contact opportunity to the active sites, thus boosting catalytic performance. Therefore, increasing the specific surface area of the MC-T series catalysts by further activation methods without decreasing the concentration of the quinone/ketone carbonyl groups seems to be essential to further enhance the DRM catalytic performance of porous carbon-based catalysts.

### 2.3. Preparation and Structural Characterization of CO_2_-Activated Catalysts

To address the fast deactivation and low H_2_/CO ratio of the MC-T series catalysts in DRM, we proposed a secondary activation strategy with CO_2_ as the activation agent. An MC-600 catalyst with a superior DRM performance was employed as the CO_2_ activation precursor. This strategy is expected to significantly increase the specific surface area and defectivity of the catalyst through carbon gasification reaction (CO_2_ + C → 2CO). Furthermore, the oxygen-rich activation environment is also beneficial for the formation of oxygen-containing functional groups, especially the quinone/ketone carbonyl groups [36]. In this section, we prepared three CO_2_-activated catalysts by changing the CO_2_ activation temperature.

**Figure 9:**
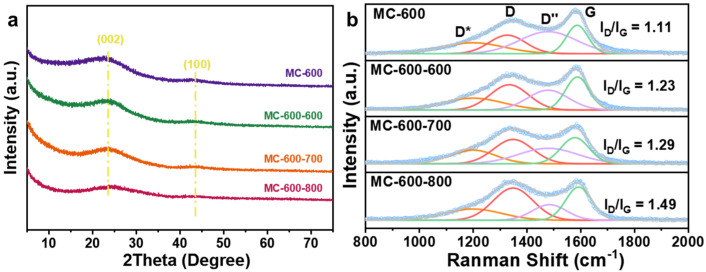
(**a**) XRD patterns and (**b**) Raman spectra of MC-600 and MC-600-600/700/800 catalysts.

Figure 9a shows the XRD patterns of MC-600 and MC-600-X (X represents the CO_2_ activation temperature). All of the CO_2_-activated catalysts showed two diffraction peaks at about 24° and 43°, which correspond to the (002) and (100) crystal planes of graphitic carbon, respectively [15]. Compared with the MC-600 catalyst, the diffraction peaks of the three modified catalysts broadened and weakened with the increase in activation temperature, indicating the increase in amorphization of the porous carbon materials catalysts [37]. The Raman spectra of the catalysts after CO_2_ activation are shown in Figure 9b. The I_D_/I_G_ of MC-600-600, MC-600-700, and MC-600-800 were 1.23, 1.29, and 1.49, respectively, which indicates that the CO_2_ activation temperature has a significant effect on the defective degree of the carbon-based catalysts. The higher the CO_2_ activation temperature, the more intense the reaction between CO_2_ and C, resulting in a higher defective degree of the catalyst [38], which is consistent with the XRD results. The defective structure could provide more anchored sites for oxygen-containing functional groups, thus enhancing the adsorption and activation of CO_2_/CH_4_ molecules as well as the DRM catalytic performance.

**Figure 10:**
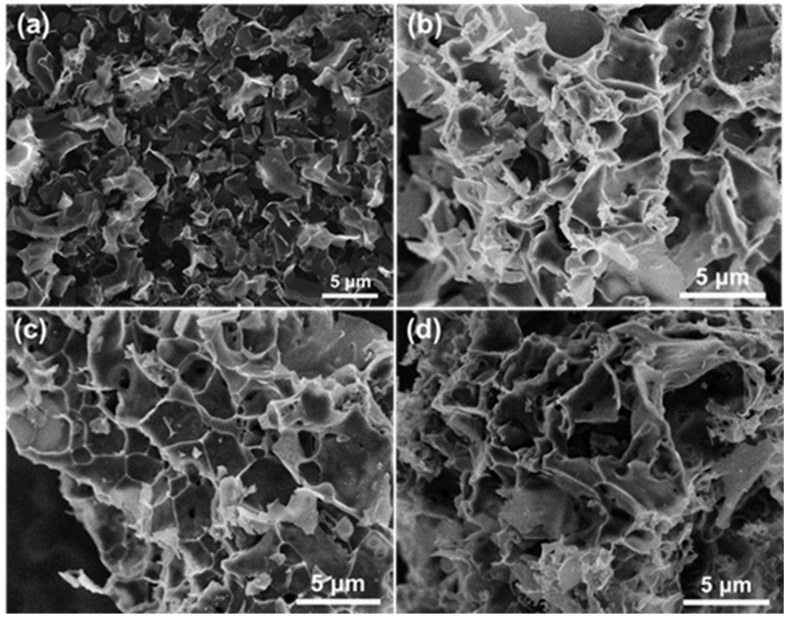
SEM images of (**a**) MC-600, (**b**) MC-600-600, (**c**) MC-600-700, and (**d**) MC-600-800 catalysts.

The microstructures of the catalysts before and after CO_2_ activations were observed by SEM. As shown in Figure 10, all catalysts showed a porous structure. Compared with the unmodified porous carbon materials catalyst MC-600, the surface of the MC-600-T series was rougher, and more pores were formed in the carbon. It has been widely accepted that the advanced architecture induced by CO_2_ activation is conducive to the enhanced transportation of reactant gases and the following DRM reaction [39].

### 2.4. Structure–Function Relationship of CO_2_-Activated Catalysts

**Figure 11:**
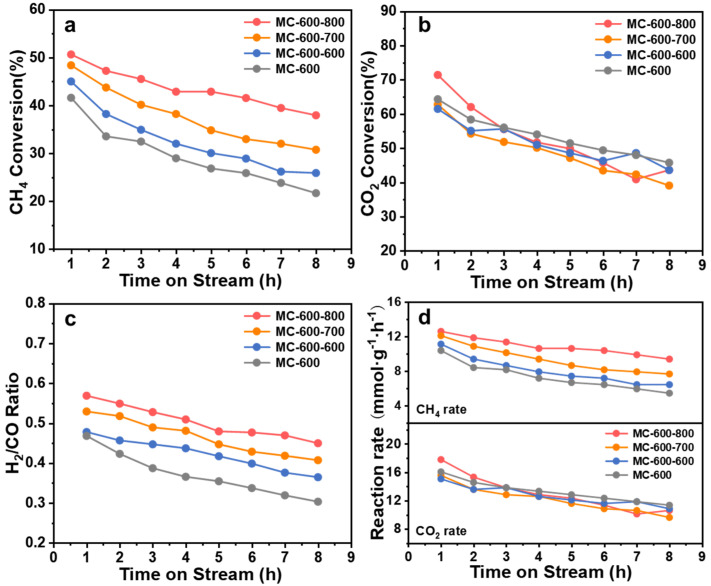
(**a**) CH_4_ conversion, (**b**) CO_2_ conversion, (**c**) H_2_/CO ratio and (**d**) Reaction rate over reaction time of MC-600 and MC-600-600/700/800 catalysts. Reaction conditions: GHSV = 6000 mL g_cat_ ^−1^ h^−1^; P = 1 atm; CH_4_: CO_2_ = 1:1; temperature: 800 °C.

The DRM performance of the CO_2_-activated catalysts was tested at 800 °C to investigate the effect of CO_2_ activation on catalyst performance (Figure 11). In general, CO_2_ activation effectively improved the CH_4_ conversion but had no significant effect on the CO_2_ conversion. The initial CH_4_ conversion of MC-600-600, MC-600-700, and MC-600-800 catalysts was 45.0%, 48.4%, and 50.7%, respectively, and the deactivation rate was lower than that of the MC-600 catalyst. It should be noted that the catalytic performance of the MC-600-600 was only slightly improved, and the enhanced DRM catalytic performance was more obvious for the MC-600-700 and MC-600-800 catalysts. The initial reaction rates reached 12.12 mmol_CH4_ g_cat._^−1^ h^−1^ and 12.62 mmol_CH4_ g_cat._^−1^ h^−1^, respectively (Figure 11d). Compared with other carbon-based metal-free catalysts, it has certain advantages under similar reaction conditions (800 °C, CH_4_/CO_2_/N_2_ = 1/1/8) (Table 4). This catalytic performance variation trend could be attributed to the architecture properties and surface quinone/ketone carbonyl group concentration on the porous carbon materials catalysts. Moreover, the CO_2_ activation step improved the H_2_/CO ratio significantly. The initial H_2_/CO ratio of MC-600-600/700/800 was 0.48, 0.53, and 0.57, respectively, which are all higher than that of the MC-600 catalyst. The CO_2_ activation might increase the number of pores and specific surface area of the catalysts, thus enhancing the adsorption of reactants.

**Figure 12:**
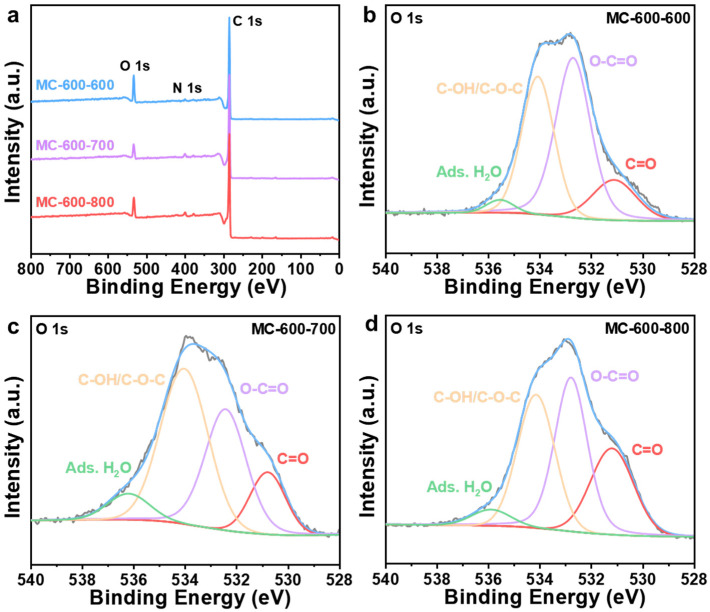
(**a**) XPS survey spectra of MC-600-600/700/800 catalysts. High-resolution O 1s XPS spectra of (**b**) MC-600-600, (**c**) MC-600-700, and (**d**) MC-600-800 catalysts.

**Figure 13:**
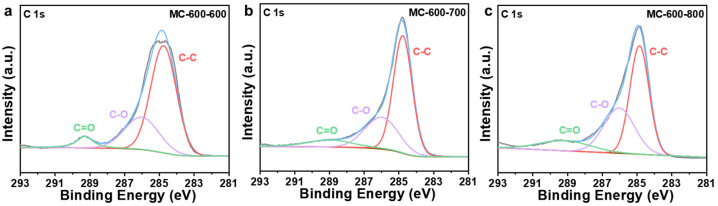
High-resolution C 1s XPS spectra of (**a**) MC-600-600, (**b**) MC-600-700, and (**c**) MC-600-800 catalysts.

XPS spectra were performed to investigate the effect of CO_2_ activation on the properties of oxygen-containing functional groups (Figure 12). After secondary activation, the catalysts were still predominantly composed of C (284.3 eV) and O (O 1s, 531.9 eV; O 2s 23.3 eV) elements [43], with a trace amount of N (399.9 eV). S (S 2p, 165 eV; S 2s, 225 eV) was also detected in the carbon-based catalyst, which can be attributed to the properties of petroleum pitch feedstock [26,27]. The N/C ratios of the three catalysts (0.03, 0.04, 0.04 for MC-600-600/700/800/, respectively) remained basically the same as the secondary activation temperature increased. In contrast, the O/C ratios of both MC-600-700 and MC-600-800 catalysts decreased compared to those of the MC-600-600 catalyst (0.32, 0.18, 0.24 for MC-600-600/700/800/, respectively). The concentration of oxygen-containing groups analyzed by the deconvolution of the O 1s XPS spectra are listed in Table 5. The relative content of the C=O functional group gradually increased as the activation temperature increased. This trend further clarified the effect of C=O on the catalytic activity of DRM [30], which is consistent with the C 1s XPS results (Figure 13, Table 6). Notably, the relative C=O contents of the MC-600-600/700/800 catalysts were lower than those of the MC-600 catalyst. The high conversions of CH_4_ and CO_2_ observed in these catalysts may be attributed to secondary activation, which resulted in a larger specific surface area and enhanced contact between the reactive gases and active sites.

**Figure 14:**
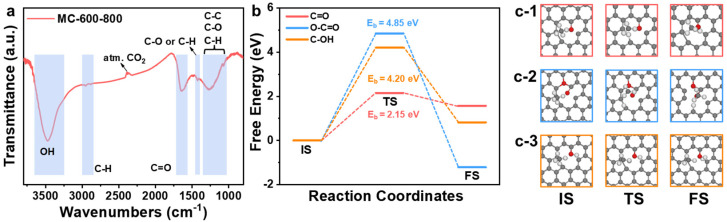
(**a**) FT-IR spectrum of MC-600-800 catalyst. (**b**) Energy profiles of CH_4_* to CH_3_* on three models. Energetically favorable configurations of CH_4_* to CH_3_* on graphene containing C=O group (**c-1**), graphene containing O-C=O group (**c-2**), and graphene containing C-OH group (**c-3**). Key: transition state (TS), E_b_ denotes the transition state energy barrier, with the C atom in grey, O atom in red, and H atom in white.

The functional groups on the surface of the MC-600-800 catalyst were monitored by Fourier Transform Infrared (FT-IR) analysis (Figure 14a). In the spectra, the stretching vibration of the -OH group was at around 3400 cm^−1^, the C-H stretching vibration peak on the benzene ring was around 3000 cm^−1^, the characteristic peak at around 2300 cm^−1^ could be attributed to atmospheric CO_2_, and the bands in the region of around 1400 cm^−1^ confirmed the existence of C-O or C-H bonds [44]. The typical peaks at around 1000–1300 cm^−1^ are usually the overlapping effect of C-C/C-O/C-H bonds, while the typical peaks at around 1600–1700 cm^−1^ can be attributed to the stretching vibration of the C=O group, which corresponds well with the XPS characterization results [44,45,46].

Considering that O-C=O also contributes to the C=O concentration, to further elucidate the effect of different oxygen-containing functional groups on the C-H bond cleavage capacity, graphene cell structures containing different oxygen-containing functional groups were constructed using Materials Studio 2018 software. This study explored the activation process of methane on these oxygen-containing functional group sites (Figure 14b,c). Density functional theory (DFT) results showed that the transition state energy barrier (2.15 eV) for the cleavage of CH_4_* to CH_3_* on the graphene-containing C=O groups was the lowest, which was much lower than that of graphene-containing O-C=O groups and C-OH groups (4.85 eV and 4.20 eV, respectively). This finding further confirmed the critical role of C=O groups in the activation of C-H bonds.

Thus, although the total content of C=O and O-C=O groups in the MC-600-600 and MC-600-800 samples is similar (61.73% and 62.42%, respectively), the C=O groups predominate in the MC-600-800 sample treated at higher temperatures. Considering that in the CH_4_ cracking reaction, the transition state barrier for C=O groups is significantly lower than that for O-C=O groups, this characteristic likely constitutes a critical factor in the superior performance of MC-600-800 over MC-600-600.

**Figure 15:**
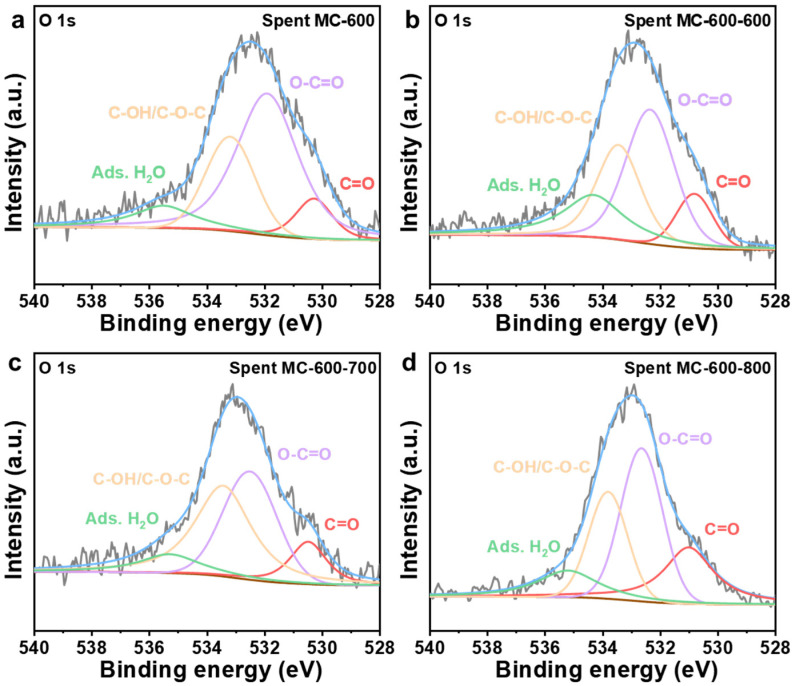
High-resolution O 1s XPS spectra of (**a**) spent MC-600, (**b**) spent MC-600-600, (**c**) spent MC-600-700, and (**d**) spent MC-600-800 catalysts.

The oxygen-containing functional groups in the catalysts after the reaction were characterized by deconvolution of the XPS O 1s spectra (Figure 15 and Table 7). After a reaction of 8 h, the relative contents of the C=O group in MC-600, MC-600-600, MC-600-700, and MC-600-800 were 9.30%, 11.60%, 12.41%, and 24.42%, respectively. Compared to the fresh counterparts, the decrease in the relative content of the C=O group in MC-600-800 throughout the reaction was unnoticeable at only 0.46%. The relative content of C=O groups in MC-600-600 and MC-600-700 decreased by 2.55% and 1.72%, respectively, during the reaction. Conversely, the MC-600 with poor stability delivered obvious C=O group degradation during the DRM reaction (from 12.90% to 9.30%). Therefore, it is reasonable to believe that the stability of the CO_2_ activated samples is closely related to the content of the C=O group (Figure 11a). This finding also implies that CO_2_ activation can improve catalyst stability by delaying the degradation of the active groups.

**Figure 16:**
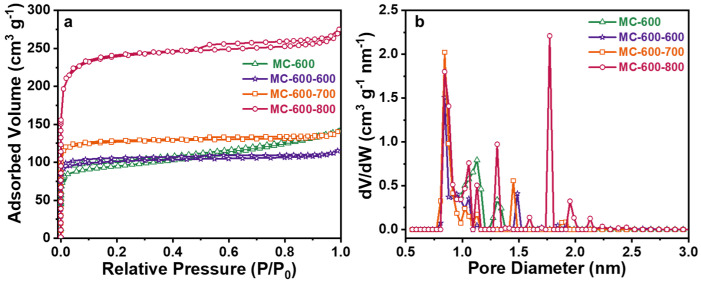
(**a**) N_2_ adsorption/desorption isotherms and (**b**) pore size distribution curves of MC-600 and MC-600-600/700/800 catalysts.

In order to compare the changes in pore structure and specific surface area of the porous carbon materials catalysts before and after CO_2_ activation, N_2_ adsorption-desorption tests were carried out (Figure 16, Table 8). From the N_2_ adsorption/desorption isotherms, as shown in Figure 12a, the adsorption curve had a steep upward slope at a relative pressure (P/P_0_) of about 0.1, which confirms the hysteretic type I N_2_ adsorption behavior [34]. The adsorption curves showed that the CO_2_-activated catalysts possessed microporous structures, indicating that the CO_2_ modification did not change the microporous properties of the MC-600 catalyst but enriched the number of micropores, as the V_t_ and V_mir_ were increased accordingly, and the V_AVE_ was reduced accordingly. With the secondary activation temperature increase, the S_BET_ of the three catalysts were 398, 500, and 917 m^2^ g^−1^, and the S_mir_ were 390, 494, and 897 m^2^ g^−1^, respectively. The S_BET_ of the CO_2_-activated catalysts were all increased compared with that of the unmodified MC-600 catalyst, with the highest increase of about 2.4 times. The pore size distribution, as shown in Figure 15b, also confirmed that the CO_2_-activated catalysts possessed more micropores than the MC-600 catalyst.

Our analyses indicate that the relative content of C=O groups and the specific surface area of the catalyst together determine the catalytic performance of the catalyst. DFT calculations also showed that C=O groups were essential in activating C-H bonds. Fortunately, due to the higher processing temperature, the O-C=O group is less stable than the C=O group, and the C=O group dominates the MC-600-800, providing it with a lower C-H cleavage energy barrier. At the same time, it has a large specific surface area, which together guarantees the excellent catalytic performance of the MC-600-800 catalyst, which coincides with the core argument of this work.

**Figure 17:**
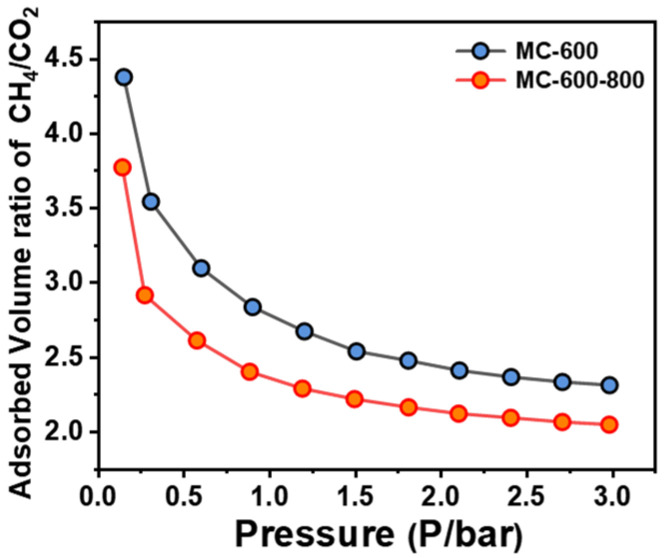
Competitive adsorption isotherms of CH_4_ and CO_2_ on MC-600 and MC-600-800 catalysts.

The adsorption behavior of reactant molecules in the pore structure of the catalysts is also crucial for the DRM performance. The impact of competitive adsorption of CO_2_ and CH_4_ molecules in the narrow pore channels on the DRM performance is inevitable. During the DRM reaction, the ideal stoichiometric ratio of CO_2_ to CH_4_ is 1, so the closer the molar content of CO_2_ and CH_4_ molecules on the porous carbon material surfaces, the more favorable for the reaction. We investigated the adsorption capacity of the MC-600 and MC-600-800 catalysts for CH_4_ and CO_2_ molecules by measuring the CH_4_/CO_2_ physisorption ratio. As shown in Figure 17, a lower gas adsorption ratio was observed on the MC-600-800 catalysts, indicating well-balanced competitive adsorption of CH_4_ and CO_2_ molecules on the active sites, which well explains their superior DRM catalytic performance.

## 3. Materials and Methods

### 3.1. Materials

Petroleum pitch (Sinopec Jiujiang Branch), anhydrous potassium carbonate (K_2_CO_3_, analytical grade), and sodium chloride (NaCl, analytical grade) were purchased from Sinopharm Chemical Reagent Group Co., Ltd., Shanghai, China. All reagents were used directly without further purification.

### 3.2. Catalyst Preparation

#### 3.2.1. Preparation of K_2_CO_3_-Activated Catalysts

Firstly, petroleum pitch (1.0 g) and K_2_CO_3_ (4.0 g) were fully grounded and mixed. Subsequently, the mixture was pyrolyzed at 500/600/700/800 °C for 2 h with a heating rate of 5 °C ⋅min^−1^ in a tube furnace under a N_2_ atmosphere. After cooling down to room temperature, the obtained product was washed with deionized water repeatedly to remove residues then collected by vacuum filtration. Finally, the obtained sample was dried in an oven at 60 °C overnight. The obtained porous carbon materials catalysts were named MC-T (T = 500, 600, 700, and 800 °C), where T represents the temperature during the calcination.

#### 3.2.2. Preparation of CO_2_-Activated Catalysts

The MC-600 sample prepared by K_2_CO_3_ activation of petroleum pitch was heated at 600/700/800 °C for 1 h with a heating rate of 5 °C min^−1^ in a tubular furnace under a CO_2_ atmosphere. The modified porous carbon materials catalysts were denoted as MC-600-600/700/800, respectively.

### 3.3. Catalytic Activity Evaluation

Dry reforming of methane was conducted in a quartz tube reactor with an inner diameter of 6 mm under atmospheric pressure. Before loading, the catalyst was crushed and sieved to 20–40 mesh. The quartz sand and catalyst particles were mixed and placed in the middle of the quartz tube reactor with quartz wool. Before the reaction, the temperature was raised to 600 °C at 5 °C min^−1^ in a N_2_ atmosphere and kept for 1 h [47] to remove impurities on the catalyst surface. At the beginning of the reaction, the catalytic performance of 10 Vol % CH_4_, 10 Vol %CO_2_, 2 Vol %N_2_ [48] (internal standard to calculate the conversion of CH_4_ and CO_2_), and 78 Vol % Ar were tested. The outlet gas was determined by a gas chromatograph (GC, Huifen HF-900, Purchased from Shandong Huifen Instrument Co., Ltd., Zaozhuang, China.) equipped with a thermal conductivity detector (TCD) and a TDX-01 column. The CH_4_ conversion, CO_2_ conversion, and H_2_/CO ratio were calculated according to the following equations (Equations (5)–(7)):

(5) $${\mathrm{CH}}_{4}\mathrm{conversation}\left(\%\right)=\frac{F_{{\mathrm{CH}}_{4},\mathrm{in}}-\;F_{{\mathrm{CH}}_{4},\mathrm{out}}}{F_{{\mathrm{CH}}_{4},\mathrm{in}}}\times 100\%$$

(6) $${\mathrm{CO}}_{2}\mathrm{conversation}\left(\%\right)=\frac{F_{{\mathrm{CO}}_{2},\mathrm{in}}-\;F_{{\mathrm{CO}}_{2},\mathrm{out}}}{F_{{\mathrm{CO}}_{2},\mathrm{in}}}\times 100\%$$

(7) $$H_{2}/\mathrm{CO}=\frac{F_{H_{2},\mathrm{out}}}{F_{\mathrm{CO},\;\mathrm{out}}}\times 100\%$$

where F_i,in_ and F_i,out_ represent the molar flow rate (mol s^−1^) of component i of the inlet and outlet.

### 3.4. Catalyst Characterization

The X-ray diffraction (XRD) patterns were evaluated using an X′ Pert Pro MPD diffractometer with Cu Kα radiation source, the scanning range was 5–75°, and the scanning rate was 10° min^−1^. The Raman spectra were conducted on a Renishaw RM2000 employing a 512 nm laser (filtered to 2.5 mW) to test the defective structure of the catalysts. The laser beam was focused on the sample surface by a 50× microscope objective. The diffraction grating and exposure time were set to 1800 l/mm and 0.1 s, respectively. The morphology and microstructure of the catalysts were observed by a scanning electron microscopy (SEM, FEI Nova nanoSEM 450) with an electron acceleration voltage of 10 kV; The specific surface area and pore structure parameters were obtained by analyzing the N_2_ adsorption/desorption isotherms, which were recorded using a Micromeritics ASAP 2020 analyzer (Micromeritics Instrument (Shanghai) Ltd., China.). Before characterization, the catalyst was degassed at 300 °C for 6 h. The specific surface area was calculated by the BET (Brunauer-EmmettTeller) equation, and other pore structure parameters were calculated by the NLDFT (Delocalized Density Function Theory) theory. The surface chemical properties, especially the type and content of oxygen-containing functional groups on the surface, were evaluated on an Escalab 250XI X-ray photoelectron spectroscopy equipped with a monochromatic Al Kα (1486.6 eV) radiation source. The obtained XPS spectra were fitted with XPS peak software (XPSPEAK 4.1) to analyze all chemical compositions. The binding energy was calibrated using the C 1s peak at 284.8 eV [49].

### 3.5. Computational Details

Materials Studio 2018 software was used to construct three graphene cell structures containing different oxygen-containing functional groups, denoted as C=O, O-C=O, and C-OH (all structures contain around 76 atoms). All structures have periodic boundary conditions.

All the computational calculations were carried out in the framework of the density functional theory (DFT) with the projector augmented plane-wave method, as implemented in the Vienna ab initio simulation package (VASP) [50,51]. The generalized gradient approximation proposed by Perdew, Burke, and Ernzerhof was employed for the exchange-correlation potential [52,53]. The cutoff energy for plane wave was set to 400 eV. Electron smearing was used via the Methfessel-Paxton technique with a smearing width consistent to σ = 0.05 eV. The Monkhorst-Pack meshes with a grid of (1 × 1 × 1) k-points were applied for all calculations. All structures were optimized until the forces on the atoms were less than 0.03 eV/Å. The transition state structure was optimized until the forces on the atoms became converged to 0.05 eV/Å. DFT-D3 functional was used to describe the van der Waals interaction [54]. All transition states were searched by combining the climbing image nudged elastic band (CI-NEB) method with the dimer method, and the stretching frequencies of the saddle points were analyzed to ensure a transition state with only one imaginary frequency [55,56].

The reaction energy (Δ*E*) and barrier (*E*_b_) were calculated via the following equations (Equations (8) and (9)):

(8) $$\Delta E=E_{\mathrm{FS}}\;-E_{\mathrm{IS}}$$

(9) $$E_{b}=E_{\mathrm{TS}}\;-E_{\mathrm{IS}}$$

where *E*_IS_, *E*_TS_, and *E*_FS_ are the total energy of the corresponding initial state (IS), transitional state (TS), and final state (FS), respectively.

## 4. Conclusions

Firstly, a series of petroleum asphalt-based derived porous carbon materials with abundant porous structures and surface oxygen-containing groups were successfully prepared using a simple K_2_CO_3_ and CO_2_ activation method and applied in metal-free catalysts for a DRM reaction. For the DRM reaction, the structure–function relationship analysis and DFT calculation showed that the quinone/ketone carbonyl (C=O) groups with the lowest CH_4_* cleavage energy barrier (2.15 eV) played critical roles in boosting the DRM reaction performance. A higher relative content of surface C=O groups could provide more active sites, thus promoting the dehydrogenation reaction of CH_4_ more effectively. When the K_2_CO_3_ activation temperature exceeds 700 °C, the C=O groups begin to decompose, and the catalyst activity decreases. By following with the secondary CO_2_ activation of the catalyst with the CO_2_ process at 800 °C, the architectural properties of the porous carbon-based catalyst were developed with the concentration of quinone/ketone carbonyl (C=O) groups well-maintained. In addition, the CO_2_ activation also effectively attenuated the competitive adsorption of CH_4_ and CO_2_ on the catalyst surface, which is necessary to boost the DRM performance. Benefiting from the cooperative interplay between the advanced architectural properties and the sufficient quinone/ketone carbonyl (C=O) groups, superior DRM performance was achieved. Surface structure of the catalyst and the content of C=O groups were significantly changed, and the contact area between the reacting gas and the active sites was increased, thus further enhancing the catalytic activity. In addition, the secondary activation also effectively attenuated the competitive adsorption of CH_4_ and CO_2_, which was necessary for enhancing the DRM performance of the metal-free carbon-based materials. The stepwise activation method proposed in this work provides a new novel strategy for modulating the surface structure and surface-active groups of carbon materials and for rationally designing metal-free porous materials as carbon-based DRM catalysts.

## Figures and Tables

**Table 1:** Surface elements and oxygen-containing groups content of MC-500/600/700/800 catalysts.

Catalysts	C=O Relative Content Percentage (%)	O=C-O Relative Content Percentage (%)	C-OH/C-O-C Relative Content Percentage (%)	Adsorbed Water (%)
MC-500	7.16	49.80	41.54	1.50
MC-600	12.90	50.79	29.66	6.65
MC-700	6.52	80.43	4.89	8.16
MC-800	8.53	32.58	52.24	6.65

**Table 2:** C-C, C-O and C=O bonds content of MC-500/600/700/800 catalysts.

Catalysts	C-C Bonds Relative Content Percentage (%)	C-O Bonds Relative Content Percentage (%)	C=O Bonds Relative Content Percentage (%)
MC-500	73.21	19.44	7.35
MC-600	60.71	25.14	14.15
MC-700	60.77	26.97	12.26
MC-800	57.96	31.10	10.94

**Table 3:** Pore structure parameters of MC-500/600/700/800 catalysts.

Catalysts	$S_{BET}^{a}$ (m^2^ g^−1^)	$S_{mic}^{b}$ (m^2^ g^−1^)	$V_{t}^{c}$ (cm^3^ g^−1^)	$V_{mic}^{d}$ (cm^3^ g^−1^)	$D_{AVE}^{e}$ (nm)
MC-500	214	170	0.13	0.09	2.40
MC-600	384	293	0.26	0.15	2.76
MC-700	849	721	0.52	0.38	2.43
MC-800	1748	1707	0.83	0.74	1.90

^a^ Calculated by BET method. ^b,d^ Calculated by t-plot method. ^c^ Total pore volume was calculated at P/P_0_ = 0.99. ^e^ Desorption average pore size.

**Table 4:** Comparison of the DRM performance obtained in this work with other literature ^a^.

Catalysts	T °C	Reaction Gas Composition	GHSV (mL g_cat._^−1^ h^−1^)	CH_4_ Conv. (%)	H_2_/CO Ratio	Reaction Rate (mmol_CH4_ g_cat._^−1^ h^−1^)	Ref.
Coal char	1050	CH_4_/CO_2_/N_2_ = 1/1/8	3750	52.7	0.75	8.15	[11]
Char	952	CH_4_/CO_2_/N_2_ = 1/1/8	1154	~50.0	~0.57	2.38	[40]
WAC	838	CH_4_/CO_2_/N_2_ = 1/1/3	6000	~53.0	~1.56	26.22	[41]
bio-char	800	CH_4_/(CO_2_+ steam) = 1/1	1200	~93.5	~2.00	23.13	[42]
Fe-C_2_	800	CH_4_/CO_2_/N_2_ = 1/1/2	2400	~97.0	~1.00	23.99	[7]
MC-600-800	800	CH_4_/CO_2_/N_2_ = 1/1/8	6000	50.7	0.57	12.62	This Work

^a^ Reaction pressure: 0.1 MPa.

**Table 5:** Surface elements and oxygen-containing groups content of MC-600-600/700/800 catalysts.

Catalysts	C=O Relative Content Percentage (%)	O=C-O Relative Content Percentage (%)	C-OH/C-O-C Relative Content Percentage (%)	Adsorbed Water (%)
MC-600-600	14.15	47.58	35.61	2.67
MC-600-700	14.13	33.91	45.18	6.78
MC-600-800	24.88	37.54	33.36	4.21

**Table 6:** C-C, C-O, and C=O bond content of MC-600-600/700/800 catalysts.

Catalysts	C-C Bonds Relative Content Percentage (%)	C-O Bonds Relative Content Percentage (%)	C=O Bonds Relative Content Percentage (%)
MC-600-600	66.30	27.33	6.37
MC-600-700	60.58	28.77	10.65
MC-600-800	51.92	35.01	13.07

**Table 7:** Surface elements and oxygen-containing groups content of spent MC-600-600/700/800 catalysts.

Catalysts	C=O Relative Content Percentage (%)	O=C-O Relative Content Percentage (%)	C-OH/C-O-C Relative Content Percentage (%)	Adsorbed Water (%)
Spent MC-600	9.30	55.80	24.30	10.60
Spent MC-600-600	11.60	40.03	27.09	21.28
Spent MC-600-700	12.41	35.25	42.06	10.28
Spent MC-600-800	24.43	37.57	24.42	13.58

**Table 8:** Pore structure parameters of MC-600 and MC-600-600/700/800 catalysts.

Catalysts	$S_{BET}^{a}$ (m^2^ g^−1^)	$S_{mic}^{b}$ (m^2^ g^−1^)	$V_{t}^{c}$ (cm^3^ g^−1^)	$V_{mic}^{d}$ (cm^3^ g^−1^)	$D_{AVE}^{e}$ (nm)
MC-600	384	293	0.26	0.15	2.76
MC-600-600	398	390	0.18	0.16	1.80
MC-600-700	500	494	0.22	0.20	1.74
MC-600-800	917	897	0.43	0.37	1.86

^a^ Calculated by BET method. ^b,d^ Calculated by t-plot method. ^c^ Total pore volume was calculated at P/P_0_ = 0.99. ^e^ Desorption average pore size.

## Data Availability

The raw data supporting the conclusions of this article will be made available by the authors on request.

## Abbreviations

AC	Activated carbon
CMD	Catalytic methane decomposition
CNT	Carbon nanotube
CB	Carbon black
DRM	Dry reforming of methane
DFT	Density-functional theory
FT-IR	Fourier transform infrared
GHSV	gas hourly space velocity
NLDFT	Non localized density functional theory
POM	Partial oxidation of methane
RWGS	Reverse water gas shift reaction
SRM	Steam reforming of methane
SEM	Scanning electron microscopy
XPS	X-ray photoelectron spectroscopy
XRD	X-ray diffraction
